# Trigeminal injury causes kappa opioid-dependent allodynic, glial and immune cell responses in mice

**DOI:** 10.1186/1744-8069-6-8

**Published:** 2010-01-29

**Authors:** Megumi Aita, Margaret R Byers, Charles Chavkin, Mei Xu

**Affiliations:** 1 Department of Pharmacology, University of Washington, Seattle, WA 98195-7280, USA; 2 Department of Anesthesiology, University of Washington, Seattle, WA 98195-7280, USA

## Abstract

**Background:**

The dynorphin-kappa opioid receptor (KOR) system regulates glial proliferation after sciatic nerve injury. Here, we investigated its role in cell proliferation following partial ligation of infraorbital nerve (pIONL), a model for trigeminal neuropathic pain. Mechanical allodynia was enhanced in KOR gene deleted mice (KOR-/-) compared to wild type mice. Using bromodeoxyuridine (BrdU) as a mitotic marker, we assessed cell proliferation in three different areas of the trigeminal afferent pathway: trigeminal nucleus principalis (Vp), trigeminal root entry zone (TREZ), and trigeminal ganglion (TG).

**Results:**

In KOR-/- mice or norBNI-treated mice, the number of proliferating cells in the Vp was significantly less than in WT mice, whereas cell proliferation was enhanced in TREZ and TG. The majority of the proliferating cells were nestin positive stem cells or CD11b positive microglia in the Vp and macrophages in the TG. GFAP-positive astrocytes made a clear borderline between the CNS and the PNS in TREZ, and phosphorylated KOR staining (KOR-p) was detectable only in the astrocytes in CNS in WT mice but not in KOR-/- or norBNI-treated mice.

**Conclusions:**

These results show that kappa opioid receptor system has different effects after pIONL in CNS and PNS: KOR activation promotes CNS astrocytosis and microglial or stem cell proliferation but inhibits macrophage proliferation in PNS. The trigeminal central root has a key role in the etiology and treatment of trigeminal neuralgia, and these newly identified responses may provide new targets for developing pain therapies.

## Background

Trigeminal nerve injury often results in debilitating chronic orofacial pain states such as trigeminal neuralgia. However, underlying mechanisms have not been completely elucidated, and the outcomes of pharmacological or surgical treatments are often disappointing [1]. To address this gap, we recently devised a mouse model of trigeminal neuropathic pain, using a partial ligation of infraorbital nerve (pIONL) that induces allodynic response to tactile stimuli within the area innervated by infraorbital nerve (ION), with substantial changes in glial proliferation and neuropeptide expression in the trigeminal ganglion and caudal medulla [2]. A strong role for the dynorphin/kappa opioid receptor (KOR) system has previously been found during responses to sciatic nerve ligation [3]. Here we use the pIONL trigeminal model to identify the role of the KOR system in mechanisms of trigeminal neuropathy.

Somatic sensory information enters the central nervous system (CNS) via the trigeminal root entry zone (TREZ), the interface between the peripheral nervous system (PNS) and CNS, where sensory axons span an environment consisting of Schwann cells in the PNS and astrocytes, oligodendrocytes and microglia in the CNS [4]. The TREZ appears to play a crucial role in trigeminal neuralgia etiology and treatment, because clinical studies have shown beneficial effects of treatments that target this area. For example, surgical microvascular decompression [5-7], glycerol rhizotomy [8] or gamma knife radiosurgery [8,9], all target the trigeminal root and entry zone. In spite of these results suggesting the importance of the TREZ in control of chronic trigeminal pain, there are very few studies characterizing the anatomical changes in this area following nerve injury.

Opioid receptors mediate the strong analgesic and addictive properties of opiate alkaloids and are the targets of endogenous opioid peptides. Our previous studies have shown that the endogenous opioid dynorphin was released following sciatic nerve injury, and sustained dynorphin release stimulated kappa opioid receptors to cause astroglial activation and proliferation in the spinal cord [3,10]. Blockade of kappa opioid system exacerbated the allodynic response and prevented astroglial activation and proliferation in spinal cord after partial sciatic nerve ligation (pSNL). Opiate alkaloids are mostly immunosuppressive *in vivo*, whereas opioid peptides such as β-endorphin and Met-enkephalin display multiple immunomodulatory effects [11,12] Opioid peptides are also involved in pain control during inflammation [13]. However, few studies have examined the influence of the kappa opioid system and blockade of kappa opioid receptor function in animal models of neuropathic pain after trigeminal nerve injury [2,14]. The current study was performed to identify the spatial pattern of cell proliferation along the nociceptive pain pathway including the trigeminal nucleus principalis (Vp), trigeminal root entry zone (TREZ) and trigeminal ganglion (TG) in comparison of wild type (WT), KOR knock out (KOR-/-) and KOR antagonist (norbinaltorphimine, norBNI) treated mice. We also examined allodynia and facial rubbing behaviors to assess the correlation between the cell proliferation pattern and the behavioral changes after pIONL.

## Results

### Behavior and general observations

#### Evoked mechanical allodynia following pIONL

**Figure 1:**
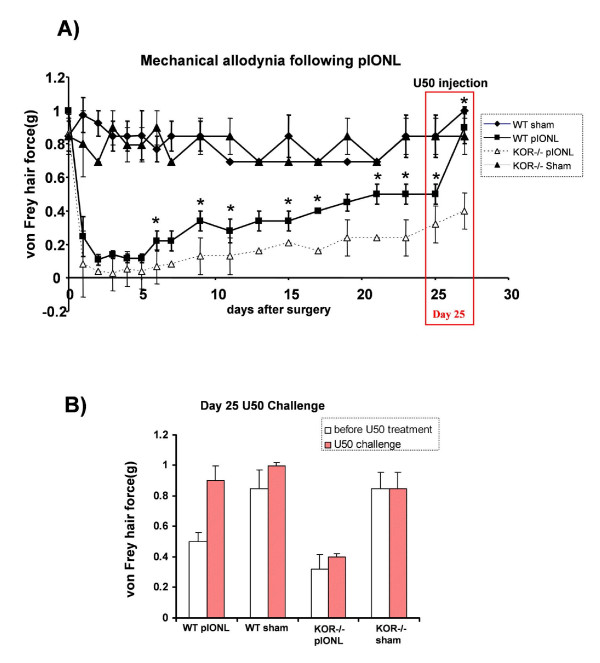
**(A) Response thresholds of ipsilateral whisker pad to usually innocuous tactile (von Frey Hair) stimuli in WT sham, WT pIONL, KOR-/- sham, and KOR-/- pIONL mice during 25 days after partial infraorbital nerve ligation**. After pIONL, both WT and KOR-/- mice developed allodynic response as evident from the decreased thresholds to tactile stimulation compared with WT and KOR-/- mice that received sham-ligation surgeries. One week after pIONL, WT mice with pIONL began to show the increase of the mechanical threshold, whereas KOR-/- pIONL group showed much slower recovery and significantly greater allodynic response than WT pIONL group. (B)On the 25^th ^day after surgery, we injected the kappa opioid receptor agonist U50,488 intraperitoneally at 10 mg/kg in each group of mice. The von Frey hair force returned to baseline in WT pIONL group, but not in KOR-/- pIONL group. U50,488 injection did not affect the mechanical threshold in sham operated groups. Asterisks indicate significant decreases in the threshold to stimuli by the von Frey hair applied to ipsilateral side of whisker pad in KOR-/- pIONL group compared to WT pIONL group (*p *< 0.05, ANOVA followed by Student-Newman test). Data are presented as the mean ± SEM von Frey hair threshold in grams; n = 8 per time point).

One day before surgery, the withdrawal threshold to mechanical stimulation of the whisker pad was measured to assess the basal threshold (Fig. 1). Before pIONL surgery, WT and KOR-/- showed similar responses to the von Frey filaments, responding with a threshold for allodynic response around 1.0 g (e.g. no allodynia). One day after surgery, the withdrawal threshold was significantly decreased on the ipsilateral side in both WT and KOR-/- mice (P < 0.05). One week after pIONL, WT mice with pIONL had significantly increased mechanical threshold, whereas KOR-/- pIONL group showed much slower recovery and significantly greater allodynic response than WT pIONL group. On the twenty fifth day after surgery, the kappa opioid receptor agonist U50,488 was injected intraperitoneally at 10 mg/kg in four experimental groups of mice (Fig. 1 B). The von Frey hair force remained at baseline for sham groups and U50,488 completely recovered the allodynia in WT pIONL group, but had no effect on allodynic responses in the KOR-/- pIONL group (as expected). These results suggest that the allodynic response induced by pIONL could be blocked by kappa opioid receptor activation.

#### Non-evoked behavior (isolated face rubbing)

**Figure 2:**
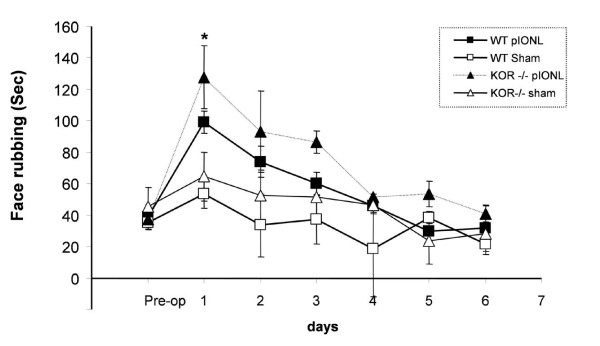
**Face rubbing after pIONL. **The total time for isolated face-rubbing episode during a 15 min observation period. The spontaneous face-rubbing behavior was recorded for 15 minutes on preoperative day 1 (baseline), and from postoperative day1 to day 6. The duration of face-rubbing behavior was elevated for both WT and KOR-/- pIONL groups on the first day after surgery compared to the baseline and sham operated groups. KOR-/- pIONL group showed longer duration of face-rubbing behavior than that of WT pIONL group on the postoperative day 1 (*p *< 0.05, ANOVA followed by Student-Newman test, n = 8 per group). The duration of face-rubbing behavior peaked at postoperative day 1 and this increased facial rubbing gradually recovered to the basal level.

The spontaneous face-rubbing behavior was video-recorded for 15 min on preoperative day 1 (baseline) and during postoperative day1 to day 6 (Fig. 2). The duration of face-rubbing behavior was elevated for both WT and KOR-/- pIONL groups on the first day after surgery compared to the baseline and sham operated groups. In addition, KOR-/- pIONL group showed longer duration of face-rubbing behavior than that of WT pIONL group on the postoperative day 1 (*p *< 0.05, ANOVA followed by Student-Newman test, n = 8 per group). The duration of face-rubbing behavior peaked at postoperative day 1. After that, facial rubbing gradually recovered to the basal level. There was no significant increase of the face-rubbing behavior in either WT or KOR-/- sham operated groups at any time point.

### Effects of KOR inhibition on astrocytic Responses

**Figure 3:**
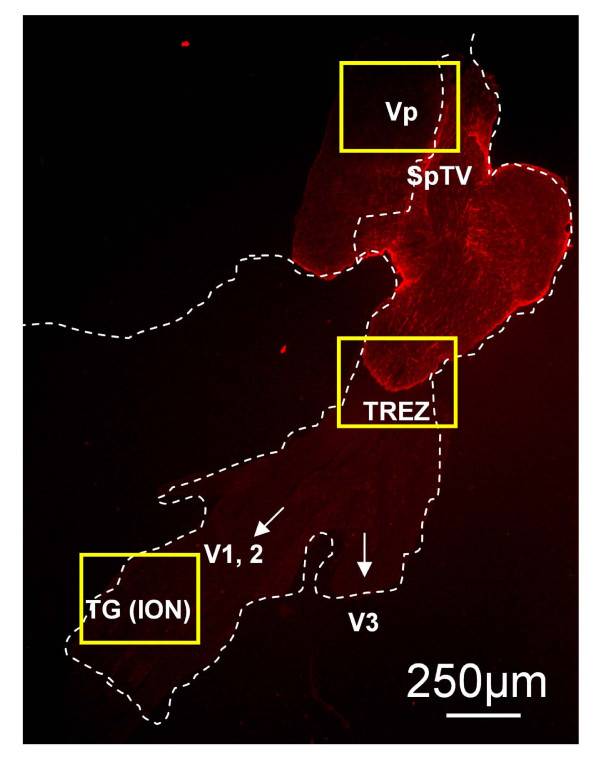
**Orientation of VP, TREZ, TG.  **Key areas for our analyses are shown here: (1) the trigeminal principle nucleus (Vp), (2) The root entry zone (TREZ) where central glia are in the CNS side (GFAP, red), and Schwann cells on the ganglion side, and (3) trigeminal ganglion (TG). The cell bodies for infraorbital nerve (ION) occur in the distal parts of the combined V1/V2 but not in V3. Yellow boxes show sites for quantification.

We measured anatomical data in Vp, TREZ and TG at 3 regions for WT mice, KOR-/- and norBNI-treated, at 8 days after pIONL, as shown Fig. 3 (GFAP staining).

#### Astrocytic response to pIONL in Vp and Trigeminal root entry zone

**Figure 4:**
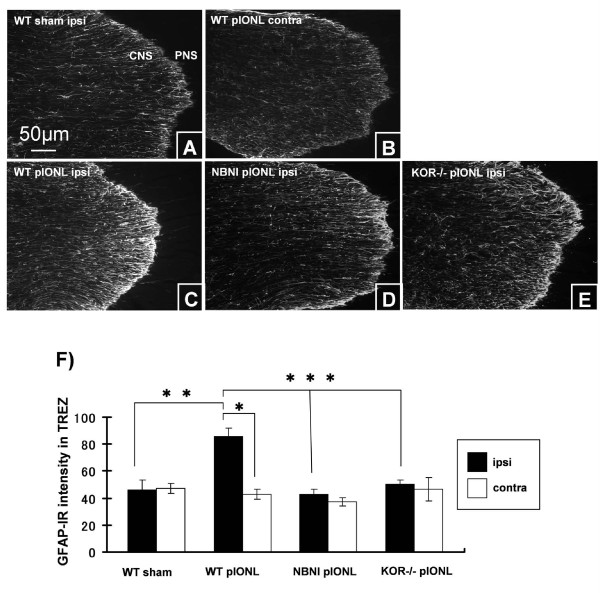
**GFAP immunoreactivity in the TREZ at day 8 after pIONL**. (A), GFAP-IR in sham operated WT mice. (B, C), pIONL induced slight increase in the intensity of GFAP staining in the ipsilateral TREZ of WT mice but not the contralateral side. (D, E), KOR-/- mice or mice pre-treated with norBNI did not show significant upregulation of GFAP immunoreactivity in the ipsilateral TREZ, and this was the same intensity as in the contralateral TREZ of WT pIONL mice (B) or WT sham operated mice(A). (F), Mean ± SEM pixel intensity of GFAP immunoreactivity in TREZ at day 8 after pIONL. (*p < 0.001) Scale bars: A-E, 50 μm

GFAP immunoreactive (IR) astrocytes were most prominent at the boundary of the Trigeminal root entry zone (TREZ), where they formed a distinct layer separating CNS and PNS tissue (Fig. 3 &4). We found that GFAP-IR increased in the ipsilateral TREZ of WT mice, especially on the CNS side immediately adjacent to the boundary at 8 days after pIONL (Fig. 4A-E) and quantification of GFAP-IR was performed within this area. Quantification showed that pIONL significantly increased the intensity of GFAP-IR in ipsilateral TREZ in WT mice compared with sham operated mice or with the contralateral TREZ (Fig. 4A, B, C, F). In contrast, neither KOR-/- nor NorBNI-treated mice showed significant changes in the expression pattern of GFAP-IR between ipsilateral and contralateral side of TREZ (Fig. 4D, E, F). Thus, the increase in the intensity of GFAP-IR astrocytes in the TREZ was blocked by the deletion of KOR or by norBNI pretreatment. Similarly, CNS astrocytosis was prominent in Vp of WT mice, but pIONL did not induce astrocytosis in KOR-/- or norBNI treated mice (data not shown).

#### KOR activation in trigeminal root entry zone

**Figure 5:**
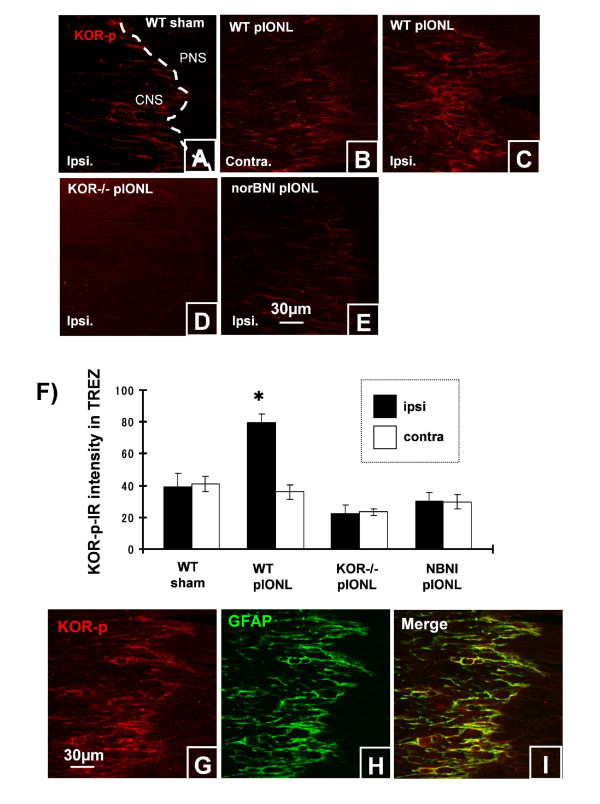
**KOR activation in TREZ at day 8 after pIONL**. (A), KOR-p-IR in sham operated WT mice. (B, C), increased KOR-p staining was seen within the root entry zone on the ipsilateral side compared to contralateral side following pIONL in WT mice. KOR activation was blocked by the deletion of kappa opioid receptor or norBNI treatment (D, E). (F), Mean ± SEM pixel intensity of KOR-p intensity in TREZ at day 8 after pIONL. (G-I), Double immunohistochemistry for KOR-p and GFAP in the TREZ at day 8 after pIONL. KOR-p staining is completely overlapped with GFAP staining. (*p < 0.001) Scale bars: A-E, 30 μm; G-I, 50 μm.

We previously established that activation of KOR results in G-protein receptor kinase mediated phosphorylation of KOR that can be readily detected using a phosphor selective antibody, KOR-p-IR [15]. Stimulation of endogenous dynorphin release can be evoked by exposure to behavioral stressors [16] or neuropathic pain [3]. These stimuli increase KOR-p-IR, and this response can be blocked by prior norBNI treatment or gene deletion of either GRK3, KOR or prodynorphin [3]. Following pIONL, the most intense KOR-p staining was localized along the CNS-PNS boundary, and KOR-p-IR intensity was measured in this area (Fig. 5A). KOR-p staining increased in the ipsilateral side compared to contralateral side 8 days following pIONL (Fig. 5B, C). KOR activation in the ipsilateral TREZ was blocked by the deletion of KOR or by NorBNI treatment (Fig. 5D, E, F). Double immunohistochemictry revealed that KOR-p-IR is expressed by astrocytes (GFAP) in TREZ of WT mice (Fig. 5G, H, I).

### Cell Proliferation along the Trigeminal Pathway

**Figure 6:**
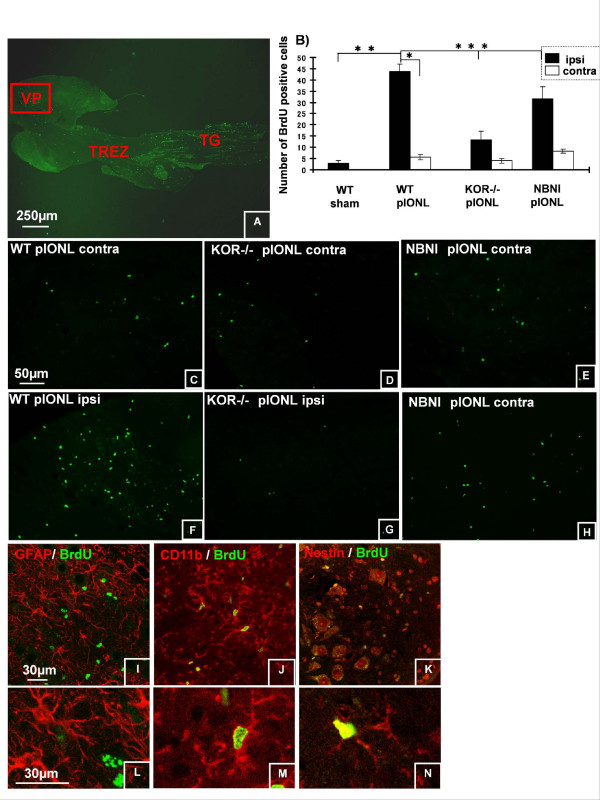
**Cell proliferation central trigeminal root at day 8 after pIONL**. (A) Data came from Vp (the area shown in red box). (B, C, F), BrdU positive cells are markedly increased in the ipsilateral Vp inWT pIONL compare to contralateral side. (B-H), in KOR-/- and NBNI pre-treated pIONL mice, the number of BrdU positive cells significantly decreased compared to WT pIONL mice. (I-N), Different cellular markers (GFAP for satellite cells, CD11b for microglia, nestin for neuronal stem cell) were used to identify the phenotypes of dividing cells. The majority of BrdU positive cells were double labeled with nestin and CD11b. Only few BrdU positive nuclei were double labeled with GFAP. (*p < 0.001) Scale bars: A, 250 μm, C-H, 50 μm, I-N, 30 μm.

**Figure 7:**
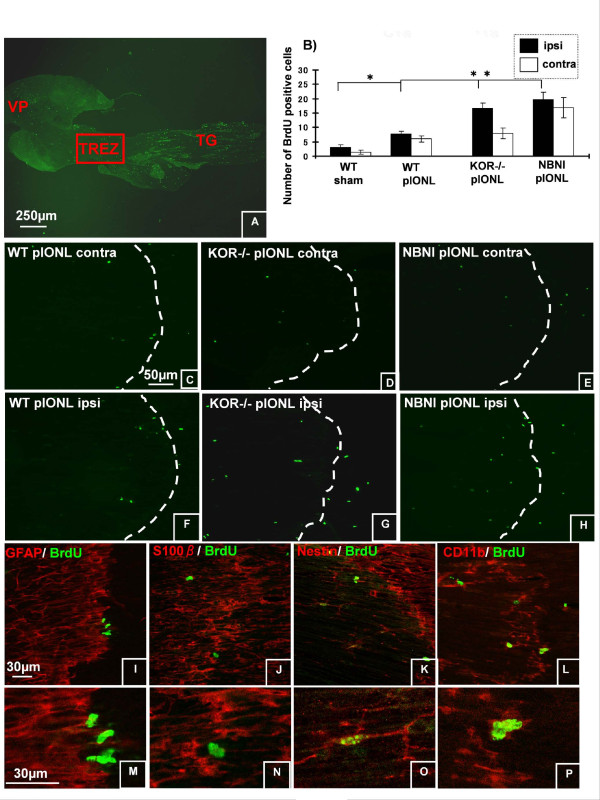
**Cell proliferation in TREZ 8 d after pIONL**. (A) Data came from TREZ (the area shown in red box). (B, C, F), the number of BrdU positive nuclei increased in both ipsilateral and contralateral TREZ following pIONL in WT compared to sham operated mice. (B-H), in the ipsilateral TREZ, KOR-/- and norBNI treated mice showed BrdU positive nuclei more than twice as much as that in WT mice. Similar to the result from Vp, the majority of BrdU positive nuclei was double-labeled with CD11b or Nestin in the CNS side of TREZ. Neither GFAP or S100beta-IR was double labeled with BrdU-IR (I-P). (*p < 0.001) Scale bars: A, 250 μm, C-H, 50 μm, I-P, 30 μm.

**Figure 8:**
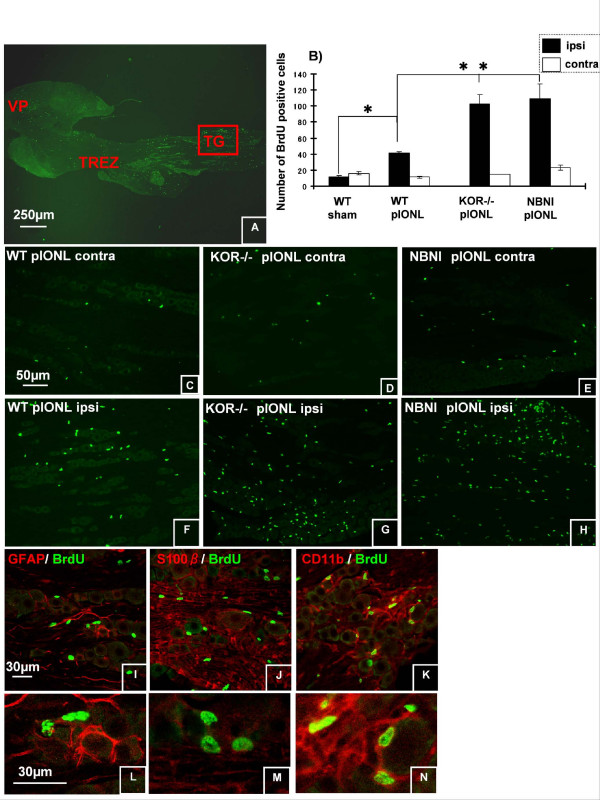
**Cell proliferation in ION region in the trigeminal ganglion at day 8 after pIONL**. (A) Data came from ION region in TG (the area shown in red box). (B, C, F), pIONL induced marked upregulation of BrdU positive cells in the ipsilateral side of ION region in WT mice. (B-H), in KOR-/- and norBNI pre-treated mice, the number of BrdU positive cells significantly increased compared to WT pIONL mice. (I-N) Different cellular markers (GFAP for satellite cells, S100beta for Schwann cells, CD11b for macrophage) were used to identify the phenotypes of dividing cells. Only rare cells were double labeled for BrdU and GFAP or S100beta. The majority of BrdU positive cells were CD11b positive macrophage. (*p < 0.001). Scale bars: A, 250 μm, C-H, 50 μm, I-N, 30 μm.

To understand the contribution of cell proliferation in trigeminal afferent pathway to neuropathic pain after pIONL, we analyzed the proliferation pattern using BrdU staining at 8 days after pIONL. We focused our analysis on three different areas: ION region in Vp (Fig. 6), the TREZ boundary between PNS and CNS (Fig. 7), and TG (Fig. 8), where we saw different expression patterns of BrdU staining.

#### Trigeminal Principal Nucleus (Vp)

In the Vp (the area is surrounded by red rectangle in Fig. 6A), the number of BrdU-IR nuclei significantly increased in ipsilateral side compared to contralateral side or sham operated mice 8 days after pIONL (Fig. 6A, B, C, F). Interestingly, the number of BrdU-IR nuclei was less in KOR-/- or norBNI treated mice than in WT (Fig. 6B, D, E, G, H). Double immunohistochemistry showed that the vast majority of BrdU-IR co-labeled with nestin (Fig. 6K, N) and CD11b (Fig. 6J, M), markers of neuronal stem cell and microglia, respectively, but not with GFAP for astrocytes (Fig. 6I, L) in this brain region.

#### Trigeminal Root Entry Zone (TREZ)

BrdU-IR did not show robust increase in TREZ and adjacent area of the border when compared to TG and Vp in WT mice (Fig. 7A, B, C, F). On the other hand, BrdU positive nuclei in KOR-/- mice or norBNI pre-treated mice showed significant increase in the ipsilateral side after pIONL (Fig. 7D, E, G, H). Double immunohistochemistry revealed that these BrdU positive nuclei were expressed by nestin positive stem cells (Fig 7K, O) and CD 11b positive microglia(Fig 7L, P) but not by either astrocytes (GFAP-IR in the CNS side, Fig 7I, M) or by Schwann cells (S100beta-IR in the PNS side, Fig 7J, N) in this area.

#### Trigeminal Ganglion (TG)

In ION region of TG(the area is surrounded by red rectangle in Fig. 8A), the number of BrdU positive nuclei robustly increased in perineuronal area compared with contralateral side or sham TG at 8 days after pIONL in WT mice (Fig. 8B, C, F). Furthermore, in the ipsilateral side of ION in KOR-/- and norBNI treated mice, BrdU-IR was nearly 2.5 times greater than that in WT (Fig. 8B, D, E, G, H). Double immunohistochemistry showed that the vast majority of these nuclei corresponded to macrophages but not satellite cells or Schwann cells because these BrdU positive nuclei were surrounded by CD11b-IR but not GFAP-IR or S100beta-IR (Fig. 8I, J, K, L, M, N).

## Discussion

The present study demonstrated that impairment of the KOR system enhances the allodynic response and delays functional recovery from partial infraorbital nerve ligation. This result is consistent with our previous study showing dynorphin KOR system regulates neuropathic pain responses following partial sciatic nerve ligation in mice [3] and shows that the spinal and trigeminal systems are similar. The current study further focused on the role of the KOR system on cell proliferation along trigeminal nociceptive pathway, the first time this has been investigated. The most novel finding of this study is that the absence of KOR activation suppressed the cell proliferation in the CNS but enhanced it in the PNS. Thus, increased allodynia in the trigeminal system may involve inhibition of CNS astrocytosis, reduced CNS microglial proliferation and enhanced macrophage invasion and proliferation in the PNS.

Previous study has shown that pIONL markedly increased the number of BrdU positive nuclei in caudal medulla of WT mice [3]. Recent studies have also indicated that orofacial nociceptive information could be more highly modulated in Vp than in other trigeminal brainstem sensory nuclei [17,18] and so we have assayed the Vp in the current work. Here, pIONL induced a marked increase of BrdU positive cells in ipsilateral Vp in WT mice and the majority of the proliferating cells were nestin positive stem cells or double-labeled by microglial marker CD11b. This result suggests that Vp is also involved in the pathological changes following pIONL. Surprisingly when KOR was impaired in knockout mice or by NorBNI treatment, proliferation of stem cells and microglia was not evident in Vp. In this region, the intensity of GFAP-IR was also decreased in KOR-/- and norBNI treated mice compared to WT mice. The consequences of this lack of the astrocyte activation would likely result in an impairment in extracellular glutamate regulation. An impairment of the glial-neuronal network that controls neuronal physiologic activities, promotes neurogenesis and secretion of neurotrophins is also probable [19-21]. A lack of the astrocyte activation might also cause the decreased number of proliferating stem cells and microglia in KOR-/- and norBNI treated mice. Together, those changes would be enhance nociceptive response after pIONL.

In the CNS compartment of TREZ in WT mice, the number of BrdU positive nuclei did not show statistically significant increase after pIONL when compared with the ipsilateral and the contralateral TREZ. [22] demonstrated that peripheral nerve injury induces cell proliferation in spinal dorsal root entry zone (DREZ) at day 2-3 following nerve surgery and after that, there was a sharp decline in the number of proliferating cells. At 7 days after surgery, cell proliferation was almost absent in DREZ. Our observation point was at day 8 after surgery and also did not show cell proliferatioin then in WT mice. In KOR-/- mice or norBNI treated mice, the number of proliferating cell was significantly greater than that in WT mice. Even in KOR-/- sham operated mice, the number of BrdU positive cells was more than that in WT pIONL mice. However, there was no significant difference between ipsilateral and contralateral TREZ in KOR-/- or norBNI treated mice. Future studies will be necessary to determine whether this greater number of proliferating cell in the TREZ in KOR-/- mice or norBNI treated mice indicate that the basal cell proliferation is higher than WT. Possibly both have an initial increase at 2-3 days after surgery, but the decline of proliferating cells is slower in KOR-/- mice than WT. In the present study, double labeling revealed that BrdU positive nuclei were expressed by microglia or nestin-expressing stem cells but not in astrocytes in the CNS compartment of TREZ. This result was consistent with previous studies [22-24]. There were also proliferating cells in the PNS compartment of TREZ, and the majority of these proliferating cells were CD11b positive macrophages.

In the trigeminal ganglion, satellite cells and Schwann cells are the predominant non-neuronal cells, but there are also macrophages that infiltrate the region following nerve injury. Previous studies have shown that cell proliferation occurs in Schwann cells [25] and in macrophages near the cell bodies of injured neurons in TG during Wallerian degeneration [26]. Schwann cells produce a number of neurotrophic factors that support the survival of injured neurons [27]. In addition, they also promote macrophage infiltration to the injured nerve [28-32] and provide a substrate for axonal growth [33-35]. In the present study, we observed increased BrdU positive cells in the ipsilateral ION region in TG at day 8 after pIONL in all injured groups. The number of proliferating cells was significantly greater in KOR-/- and norBNI treated mice than that in WT mice, suggesting that the lack of kappa opioid system enhanced cell proliferation in the TG. Double immunohistochemistry showed that these BrdU positive cells were all expressed in CD11b positive macrophages. Increase in the density of macrophages in DRGs after peripheral nerve lesions has previously been described [36-39] and that has been shown to contribute to neuronal survival in the short term and generation of chronic neuropathic pain by triggering neuronal impulses.

The endogenous kappa selective peptide, dynorphin, has been shown to increase macrophage superoxide production [40], modulate macrophage oxidative burst [41], enhance macrophage tumoricidal activity [42,43] and increase production of the cytokine IL-1 from bone marrow macrophages [44]. Both T cells and macrophages are targets for kappa agonists to produce inhibition of T-cell-mediated antibody production [12,45,46]. Both endogenous and exogenous opioids have been found to modulate the immune response [47]. In many cases, treatment with opioids results in an impairment of immune function [11]. In addition, previous study has shown that resting macrophage phagocytic activity is depressed by treatment with *μ*-opioid-, *δ*-opioid-, and *κ*-opioid-selective agonists, and that the inhibitory activities of these compounds can be reversed by the corresponding opioid-type-selective antagonist [48]. These studies suggest that kappa opioid receptors on macrophages are involved in cell proliferation after peripheral nerve injury. The kappa opioid system may prevent the expansion of neuro-inflammation by controlling the activation of macrophage.

In this study, we used an antibody which detects the phosphorylated form of kappa opioid receptor to determine the sites of endogenous dynorphin action in TG, TREZ and Vp. Previous studies [15,16,3] have shown that the sustained release of endogenous dynorphins during chronic pain increased KOR phosphorylation as detected by KOR-p staining. We found that the upregulation of KOP-p staining was seen only in the CNS side of the TREZ and only in WT mice. The intensity of KOR-p staining increased for the ipsilateral side of TREZ and was blocked by the deletion of kappa opioid receptor or pre-treatment with kappa opioid receptor antagonist norBNI at 8 days after pIONL. Consistent with our previous studies, KOR-p staining was co-localized with GFAP staining in the CNS side of TREZ. GFAP staining showed a similar pattern of KOR-p staining, which was upregulated in ipsilateral TREZ in WT mice after pIONL, and this upredulated GFAP was not evident in KOR-/- and norBNI treated mice. We did not find kappa opioid receptor activation in the ganglion at day 8 after pIONL. However, this result does not automatically indicate that kappa opioid receptor does not exist in trigeminal nerve or ganglia. The density of kappa opioid receptor is low in immune cells and only small subpopulations of immune cells may express the receptor. The cell proliferative patterns described in the various CNS and PNS locations show not only interactions between the injured neurons but also between non-neuronal cells residing in the affected regions. Furthermore, our results suggested that endogenous kappa opioid system modulates the proliferation of immune cell in a different manner between PNS and CNS, and the lack of the kappa opioid receptor leads to enhanced allodynic response after peripheral nerve injury.

## Conclusions

These results show that kappa opioid receptor system has different effects after pIONL in CNS and PNS: KOR activation promotes CNS astrocytosis and microglial or stem cell proliferation but inhibits macrophage proliferation in PNS. Our demonstration of the importance of the endogenous kappa opioid system for the activation of astrocytes and proliferation of stem cells and immune cells after peripheral nerve injury helps to understand the neuroinflammatory changes in the trigeminal system using a mouse model that causes persistent trigeminal allodynia. The lack of astrocyte proliferation at the CNS sites differs from CNS responses to sciatic nerve injury [10]. This work provides new targets for development of KOR modulating drugs for regulation of the immune system and inflammatory reactions after peripheral nerve injury in general, and for trigeminal neuropathic pain in particular.

## Materials and methods

### Animals

Male C57Bl/6 mice (Charles River Laboratories, Wilmington, MA) weighing 22-32 g (12-16 weeks old) were used in these experiments. Mice were group-housed, in self-standing plastic cages (28 cm L × 16 cm W × 13 cm H) within the animal core facility at the University of Washington, and maintained in a specific pathogen-free housing unit. Mice were transferred 1 week prior to behavior testing into a colony room adjacent to the testing room to acclimatize to the testing environment. Housing rooms were illuminated on a 12-h light-dark cycle with lights on at 0700. Food pellets and water were available *ad libitum*. Procedures with mice were approved by the Institutional Animal Care and Use Committee in accordance with the 1996 NIH Guide for the Care and Use of Laboratory Animals.

### Surgical preparation: Partial Ligation of the Infraorbital Nerve (pIONL)

As previously described [2], the unilateral partial ligation to the right ION was performed under direct visual control using a Zeiss surgical microscope (×10-25). The animals were anesthetized with sodium pentobarbital (Nembutal, 80 mg/kg i.p.). They were kept warm with a heat lamp and foil blanket, their eyes were treated with lubricating ophthalmic ointment (Akorn, Buffalo Grove, IL), and the top of the snout was shaved and rubbed with iodine. The mouse was taped to a sterilized cork board, the skin along the top of the snout was shaved and iodine treated, and a mid-line incision was made to expose nasal and maxillary bone. All tools were gas sterilized prior to surgery and then washed and heat-treated (glass beads at 250°C) between animals. The left ION was initially exposed 1-2 mm rostral to infraorbital foramen on the maxillary bone using blunt dissection with small scissors. The ION was gently isolated using fine forceps without damaging nearby facial nerve branches. Approximately 1/3 to 1/2 the diameter of the nerve was tightly ligated with 7-0 silk suture (Surgical Specialties Corporation, Reading, PA) by passing the suture needle completely under the lateral aspect of the nerve and then up through the middle. After confirming hemostasis, the incision was closed using silk sutures (5-0). For the sham-operated mice, the ION was exposed on the left side using the same procedure without damaging ION. The mice received one analgesic (buprenorphine, 0.05 mg/kg) treatment at the end of the surgery. The operated mice were able to eat and drink unaided soon after waking up, the body weight returned back to or exceeded preoperative weights after the first week.

### Behavioral testing

#### Stimulus-evoked responses: Mechanical allodynia

The mice were tested one day before surgery, daily during the first postoperative week, and on alternate days after that. All experiments were carried out in a quiet room between 0800 and 1500 hr. Body weight was measured every time before testing. On the day of testing, mice were habituated to handling and testing equipment 20-30 min before experiments. A graded series of von Frey filaments (Semmes-Weinstein monofilaments, Stoelting, Wood Dale, IL) was used for mechanical stimulation of ipsilateral infraorbital nerve territory. The filaments produced a bending force of 0.02, 0.04, 0.07, 0.16, 0.4, 0.6, 1.0 and 1.4 gm. The mice stood on a metal mesh with a porous plastic cup (diameter: 8 cm) covering them. Von Frey hairs were then inserted from below through the mesh. The stimuli were applied within the infraorbital nerve territory, near the center of the vibrissa pad, on the hairy skin of the ipsilateral side. The response threshold was determined by a brisk withdrawal of the head. The stimulation always began with the filament producing the lowest force and stopped when threshold was found. Unresponsive mice received a maximum stimulus score of 1.4 gm.

#### Non-evoked Behavior: Facial rubbing behavior

Mice were placed individually in small transparent plastic cages (14 cm × 16 cm × 13 cm) without bedding. A video camera was placed 0.8 m at the side of the cage and positioned so that the image of the mouse head was observed. Mice were habituated in the cage for 15 min and then recorded 15 min per day. Duration of face rubbing actions was recorded as the total time that the forelimbs contacted facial region and ears.

### Immunohistochemistry

Mice were anesthetized with sodium pentobarbital (100 mg/kg i.p.) and intracardially (i.c.) perfused with 4% para-formaldehyde in PB (phosphate buffer, 0.1 M sodium phosphate, pH 7.4). The trigeminal ganglia (TG) were dissected, postfixed for 2 hours, cryoprotected with solution of 30% (w/v) sucrose in PB at 4°C overnight and cut into series of 30 μm sections (brainstem) or 10 μm serial sections (TG plus TREZ and attached principal nucleus region of brainstem) with a cryostat microtome. TG sections were mounted onto Supefrost/Plus slide glass (Fisher Scientific) and frozen at -20°C until use. Sections were washed 3 times in 0.1 M PBS (phosphate buffer saline, pH7.4), blocked in PBS containing 0.1% Triton X-100 and 5% normal goat serum for 1 hr, and incubated overnight with primary antibodies. Primary antibody concentrations were as follows: rabbit anti-phosphorylated kappa opioid receptor (20 μg/ml) generated as previously described (McLaughlin et al., 2004), rat anti-CD11b (1:200, Serotec, Oxford, UK), rabbit anti-GFAP (1:1000, Dako, Denmark), mouse anti-nestin (Abcam, Cambridge, MA) and mouse anti-BrdU (6 μg/ml, Millipore, Temecula CA) or rat anti-BrdU (1: 50 Abcam Cambridge, MA). For BrdU staining, sections were treated with 2N HCl for 60 min at 37°C, followed by rinsing in 0.1 M borate buffer before incubation overnight with primary antibody. Sections were then washed with PBS, and detection was carried out using the rhodamine (TRITC) or fluorescein (FITC) conjugated fluorescent secondary antibodies (1:250; Jackson ImmunoResearch, West Grove, PA). Antibodies were diluted in a solution containing 0.1% Triton X-100 and 1.5% normal goat serum in PBS. The finished sections were rinsed in PBS for 30 min, and then mounted on Superfrost/Plus slide glass (Fisher Scientific) with Vectashield mounting medium (Vector Laboratories, Burlingame, CA) and sealed with nail polish. The sections were viewed with a Nikon Eclipse E600 fluorescence microscope (Tokyo, Japan) or a Leica SL confocal microscope located in the W.M. Keck Imaging Facility at the University of Washington.

### Chemicals (Drugs Sources, Dosage, Timing)

NorBNI was obtained from the National Institute on Drug Abuse drug supply program (National Institutes of Health, Bethesda, MD). NorBNI is an antagonist for KOR and it was given at 10 mg/kg (i.p.). It is active for a week, but uniformity of dosage is best achieved by giving the drug every 3 days (McLaughlin et al., 2003). U50,488 was obtained from Sigma, St. Louis, Mo USA. U50,488 is an agonist for KOR and it was given at 10 mg/kg (i.p.) 30 minutes before behavioral tests were done. The doses of NorBNI and U50,488 were chosen based on our previous publication (Xu et al., 2004). 5-Bromo-2'-deoxyuridine (BrdU) was from Sigma (St. Louis, MO). Mice were injected intraperitoneally with BrdU solution in saline (100 mg of BrdU per kilogram of body weight) once a day for 7 days starting from the day of partial infraorbital nerve ligation. BrdU is a thymidine analog that is specifically incorporated into DNA during DNA synthesis that can be used as a marker for proliferation.

### Data Analysis

Mechanical allodynia data were analyzed by ANOVA. For all groups, pre- and post-operative behavior for intra-animal comparisons as well as group comparisons were made. Statistical significance determined by ANOVA was then further analyzed with Student-Newman-Keuls test or Student's *t *test for significant pair-wise comparisons. Response data are presented as means ± SEM of the animal treatment group, with significance set at *p *< 0.05. Estimations of the total number of nuclei positive for BrdU was calculated at an objective magnification of ×20 in an ocular frame (area 0.34 mm^2^). In order to count cells in the three different areas, the frame was oriented in the following ways. The trigeminal tract: the frame was oriented so that the thin line of Lissauer's tract and the adjacent grey matter were included. TREZ: the frame was placed so that the boundary of CNS and PNS and the adjacent PNS region were included. ION: the frame was placed in the ION region, which was confirmed by our previous study using ATF3 expression [2]. Altogether, counts were made in at least 6 complete frames from each area and each animal. The averaged numbers of BrdU in all the frames within each tissue compartment were calculated. The quantitative data on numbers of proliferating cells following pIONL was analysed with student's *t*-test for each area with significance set at *p *< 0.05.

The density of immunoreactivity on each slide of TG was evaluated using NIH image J. For structures in which immunoreactivity was upregulated after nerve injury, mean optical density (brightness) was determined after subtracting background grey levels. Data were reported as means ± SEM. Statistical differences (*p *< 0.05) were determined by t-test.

## Competing interests

The authors declare that they have no competing interests.

## Authors' contributions

AM carried out the immunoassays and participated in writing the manuscript. MB and CC participated in the design of the study, helped write some sections and made critical comments on manuscript drafts. MX coordinated the project, carried out the behavioral tests and prepared the final version of the manuscript. All authors read and approved the final manuscript.

## References

[B1] Cheshire WP Trigeminal neuralgia: for one nerve a multitude of treatments Expert Rev Neurother 2007 7 1565 1579 10.1586/14737175.7.11.1565 17997704

[B2] Xu M Aita M Chavkin C Partial infraorbital nerve ligation as a model of trigeminal nerve injury in the mouse: behavioral, neural, and glial reactions J Pain 2008 9 1036 1048 10.1016/j.jpain.2008.06.006 18708302 PMC2632609

[B3] Xu M Petraschka M McLaughlin JP Westenbroek RE Caron MG Lefkowitz RJ Czyzyk TA Pintar JE Terman GW Chavkin C Neuropathic pain activates the endogenous kappa opioid system in mouse spinal cord and induces opioid receptor tolerance J Neurosci 2004 24 4576 4584 10.1523/JNEUROSCI.5552-03.2004 15140929 PMC2376823

[B4] Toma J McPhail LT Ramer MS Comparative postnatal development of spinal, trigeminal and vagal sensory root entry zones Int J Devel Neurosci 2006 24 373 388 10.1016/j.ijdevneu.2006.06.001 16911863

[B5] Jannetta PJ Abbasy M Maroon JC Ramos FM Albin MS Etiology and definitive microsurgical treatment of hemifacial spasm. Operative techniques and results in 47 patients J Neurosurg 1977 47 321 8 10.3171/jns.1977.47.3.0321 894338

[B6] McLaughlin MR Jannetta PJ Clyde BL Subach BR Comey CH Resnick DK Microvascular decompression of cranial nerves: lessons learned after 4400 operations J Neurosurg 1999 91 1 8 10.3171/jns.1999.91.1.0001 10413149

[B7] Li ST Wang X Pan Q Hai J Liu N Shen F Liu Z Guan Y Studies on the operative outcomes and mechanisms of microvascular decompression in treating typical and atypical trigeminal neuralgia Clin J Pain 2005 21 311 316 10.1097/01.ajp.0000120790.69705.5b 15951648

[B8] Henson CF Goldman HW Rosenwasser RH Downes MB Bednarz G Pequignot EC Werner-Wasik M Curran WJ Andrews DW Glycerol rhizotomy versus gamma knife radiosurgery for the treatment of trigeminal neuralgia: an analysis of patients treated at one institution Int J Radiat Oncol Biol Phys 2005 63 82 90 16111575 10.1016/j.ijrobp.2005.01.033

[B9] McNatt SA Gonzalez-Gomez I Nelson MD McComb JG Gamma knife radiosurgery for trigeminal neuralgia Neurology 2005 56 1295 1303 10.1227/01.neu.0000160073.02800.c7 15918946

[B10] Xu M Bruchas MR Ippolito DL Gendron L Chavkin C Sciatic nerve ligation-induced proliferation of spinal cord astrocytes is mediated by kappa opioid activation of p38 mitogen-activated protein kinase J Neurosci 2007 27 2570 2581 10.1523/JNEUROSCI.3728-06.2007 17344394 PMC2104780

[B11] Bryant HU Roudebush RE Suppressive effects of morphine pellet implants on in vivo parameters of immune function J Pharmacol Exp Ther 1990 255 410 4 2243332

[B12] Eisenstein TK Hilburger ME Opioid modulation of immune responses: effects on phagocyte and lymphoid cell populations J Neuroimmumol 1998 83 36 44 10.1016/S0165-5728(97)00219-1 9610671

[B13] Machelska H Cabot PJ Mousa SA Zhang Q Stein C Pain control in inflammation governed by selectins Nat Med 1998 4 1425 1428 10.1038/4017 9846582

[B14] Schmidt BL Tambeli CH Levine JD Gear RW mu/delta Cooperativity and opposing kappa-opioid effects in nucleus accumbens-mediated antinociception in the rat Eur J Neurosci 2002 15 861 868 10.1046/j.1460-9568.2002.01915.x 11906528

[B15] McLaughlin JP Myers LC Zarek PE Caron MG Lefkowitz RJ Czyzyk TA Pintar JE Chavkin C Prolonged kappa opioid receptor phosphorylation mediated by G-protein receptor kinase underlies sustained analgesic tolerance J Biol Chem 2004 279 1810 1818 10.1074/jbc.M305796200 14597630 PMC2131729

[B16] McLaughlin JP Xu M Mackie K Chavkin C Phosphorylation of a carboxyl-terminal serine within the kappa-opioid receptor produces desensitization and internalization J Biol Chem 2003 278 34631 40 10.1074/jbc.M304022200 12815037

[B17] Bae YC Nakagawa S Yoshida A Nagase Y Takemura M Shigenaga Y Morphology and synaptic connections of slowly adapting periodontal afferent terminals in the trigeminal subnuclei principalis and oralis of the cat J Comp Neurol 1994 348 121 132 10.1002/cne.903480107 7814681

[B18] Kim YS Paik SK Cho YS Shin HS Bae JY Moritani M Yoshida A Ahn DK Valtschanoff J Hwang SJ Moon C Bae YC Expression of P2X3 receptor in the trigeminal sensory nuclei of the rat J Comp Neurol 2008 506 627 39 10.1002/cne.21544 18067147

[B19] Gray CW Patel AJ Neurodegeneration mediated by glutamate and beta-amyloid peptide: a comparison and possible interaction Brain Res 1995 691 169 179 10.1016/0006-8993(95)00669-H 8590049

[B20] Araque A Parpura V Sanzgiri RP Haydon PG Glutamate-dependent astrocyte modulation of synaptic transmission between cultured hippocampal neurons Eur J Neurosci 1998 10 2129 2142 10.1046/j.1460-9568.1998.00221.x 9753099

[B21] Schwartz JP Nishiyama N Neurotrophic factor gene expression in astrocytes during development and following injury Brain Res Bull 1994 35 403 407 10.1016/0361-9230(94)90151-1 7532097

[B22] Liu L Rudin M Kozlova EN Glial cell proliferation in the spinal cord after dorsal rhizotomy or sciatic nerve transection in the adult rat Exp Brain Res 2000 131 64 73 10.1007/s002219900273 10759172

[B23] Narita M Yoshida T Nakajima M Narita M Miyatake M Takagi T Yajima Y Suzuki T Direct evidence for spinal cord microglia in the development of a neuropathic pain-like state in mice J Neurochem 2006 97 1337 1348 10.1111/j.1471-4159.2006.03808.x 16606373

[B24] Echeverry S Shi XQ Zhang J Characterization of cell proliferation in rat spinal cord following peripheral nerve injury and the relationship with neuropathic pain Pain 2008 135 37 47 10.1016/j.pain.2007.05.002 17560721

[B25] Thomas GA Quantitative histology of Wallerian degeneration: II. Nuclear population in two nerves of different fibre spectrum J Anat 1948 82 135 145 PMC1273009 18873156

[B26] Hu P Bembrick AL Keay KA McLachlan EM Immune cell involvement in dorsal root ganglia and spinal cord after chronic constriction or transection of the rat sciatic nerve Brain Behav Immun 2007 21 599 616 10.1016/j.bbi.2006.10.013 17187959

[B27] Scherer SS Salzer J Axon-Schwann cell interaction during peripheral nerve degeneration and regeneration, Glial Cell Development 2003 Oxford University Press, London 299 330

[B28] Banner LR Patterson PH Major changes in the expression of the mRNAs for cholinergic differentiation factor/leukemia inhibitory factor and its receptor after injury to adult peripheral nerves and ganglia Proc Natl Acad Sci USA 1994 91 7109 13 10.1073/pnas.91.15.7109 8041754 PMC44348

[B29] Bolin LM Verity AN Silver JE Shooter EM Abrams JS Interleukin-6 production by Schwann cells and induction in sciatic nerve injury J Neurochem 1995 64 850 858 7830079 10.1046/j.1471-4159.1995.64020850.x

[B30] Siebert H Sachse A Kuziel WA Maeda N Bruck W The chemokine receptor CCR2 is involved in macrophage recruitment to the injured peripheral nervous system J Neuroimmunol 2000 110 177 185 10.1016/S0165-5728(00)00343-X 11024548

[B31] Toews AD Barrett C Morell P Monocyte chemoattractant protein 1 is responsible for macrophage recruitment following injury to sciatic nerve J Neurosci Res 1998 53 260 267 10.1002/(SICI)1097-4547(19980715)53:2<260::AID-JNR15>3.0.CO;2-A 9671983

[B32] Tofaris GK Patterson PH Jessen KR Mirsky R Denervated Schwann cells attract macrophages by secretion of leukemia inhibitory factor (LIF) and monocyte chemoattractant protein-1 in a process regulated by interleukin-6 and LIF J Neurosci 2002 22 6696 6703 12151548 10.1523/JNEUROSCI.22-15-06696.2002 PMC6758146

[B33] Araki T Milbrandt J Ninjurin, A novel adhesion molecule, is induced by nerve injury and promotes axonal growth Neuron 1996 17 353 61 10.1016/S0896-6273(00)80166-X 8780658

[B34] Kleitman N Wood P Johnson MI Bunge RP Schwann cell surfaces but not extracellular matrix organized by Schwann cells support neurite outgrowth from embryonic rat retina J Neurosci 1988 8 53 663 10.1523/JNEUROSCI.08-02-00653.1988 PMC6569303 3339432

[B35] Martini R Expression and functional roles of neural cell surface molecules and extracellular matrix components during development and regeneration of peripheral nerves J Neurocytol 1994 23 1 28 10.1007/BF01189813 8176415

[B36] Gehrmann J Monaco S Kreutzberg GW Spinal cord microglial cells and DRG satellite cells rapidly respond to transection of the rat sciatic nerve Restor Neurol Neurosci 1991 2 181 198 10.3233/RNN-1991-245605 21551602

[B37] Lu X Richardson PM Responses of macrophages in rat dorsal root ganglia following peripheral nerve injury J Neurocytol 1993 22 334 341 10.1007/BF01195557 8315414

[B38] Hu P McLachlan EM Macrophage and lymphocyte invasion of dorsal root ganglia after peripheral nerve lesions in the rat Neuroscience 2002 112 23 38 10.1016/S0306-4522(02)00065-9 12044469

[B39] Smith ML Adrian EK On the presence of mononuclear leucocytes in dorsal root ganglia following transection of the sciatic nerve Anat Rec 1972 172 581 588 10.1002/ar.1091720311 5011947

[B40] Sharp BM Keane WF Suh HJ Gekker G Tsukayama D Peterson PK Opioid peptides rapidly stimulate superoxide production by human polymorphonuclear leukocytes and macrophages Endocrinology 1985 117 793 795 10.1210/endo-117-2-793 2862014

[B41] Tosk JM Grim JR Kinback KM Sale EJ Bozetti LP Will AD Modulation of chemiluminescence in a murine macrophage cell line by neuroendocrine hormones Int J Immunopharmacol 1993 15 615 620 10.1016/0192-0561(93)90079-E 8104166

[B42] Foster JS Moore RN Dynorphin and related opioid peptides enhance tumoricidal activity mediated by murine peritoneal macrophages J Leuk Biol 1987 42 171 174 10.1002/jlb.42.2.171 2439627

[B43] Hagi K Uno K Inaba K Muramatsu S Augmenting effect of opioid peptides on murine macrophage activation J Neuroimmunol 1994 50 71 76 10.1016/0165-5728(94)90216-X 7905488

[B44] Apte RN Durum SK Oppenheim JJ Opioids modulate interleukin-1 production and secretion by bone-marrow macrophages Immunol Lett 1990 24 141 148 10.1016/0165-2478(90)90026-M 2162329

[B45] Guan L Townsend R Eisenstein TK Adler MW Rogers TK Both T-cells and macrophages are target of κ-opioid-induced immunosuppression Brain Behav Immun 1994 8 229 240 10.1006/brbi.1994.1021 7865894

[B46] Guan L Eisenstein TK Adler MW Rogers TJ Inhibition of T cell superantigen responses following treatment with the kappaopioid agonist U50, 488H J Neuroimmunol 1997 75 163 168 10.1016/S0165-5728(97)00018-0 9143250

[B47] Wybran J Appelboom T Famaey JP Govaerts A Suggestive evidence for receptors for morphine and methionine-enkephalin on normal human blood T lymphocytes J Immunol 1979 123 1068 1070 224107

[B48] Szabo I Rojavin M Bussiere JL Eisenstein TK Adler MW Rogers TJ Suppression of peritoneal macrophage phagocytosis of *Candida albicans *by opioids J Pharmacol Exp Ther 1993 267 703 706 8246144

